# Survival in Liver Transplant Recipients with Hepatitis B- or Hepatitis C-Associated Hepatocellular Carcinoma: The Chinese Experience from 1999 to 2010

**DOI:** 10.1371/journal.pone.0061620

**Published:** 2013-04-16

**Authors:** Zhenhua Hu, Jie Zhou, Haibo Wang, Min Zhang, Shaogang Li, Yuzhou Huang, Jian Wu, Zhiwei Li, Lin Zhou, Shusen Zheng

**Affiliations:** 1 Department of Hepatobiliary and Pancreatic Surgery, First Affiliated Hospital, School of Medicine, Zhejiang University, Hangzhou, China; 2 Key Laboratory of Combined Multi-Organ Transplantation, Ministry of Public Health, First Affiliated Hospital, School of Medicine, Zhejiang University, Hangzhou, China; 3 Key Laboratory of Organ Transplantation, First Affiliated Hospital, School of Medicine, Zhejiang University, Hangzhou, China; 4 China Liver Transplant Registry, Hongkong, China; The University of Hong Kong, Hong Kong

## Abstract

**Background:**

Hepatitis B virus-associated hepatocellular carcinoma (HBV-HCC) and hepatitis C virus (HCV)-HCC are the main indications for liver transplantation. We compared differences in survival outcomes between these two conditions.

**Methods and Findings:**

The China Liver Transplant Registry (CLTR) contains data collated from all transplants performed in 86 liver transplantation centers across China. We analyzed CLTR data from January 1999 to December 2010. In all, 7,658 patients (7,162 with HBV-HCC and 496 with HCV-HCC) were included in this study. Clinical characteristics were compared between the HBV-HCC and HCV-HCC groups; Kaplan–Meier analysis was used to calculate the overall, tumor-free and hepatitis-free survival rates. The 1-year, 3-year and 5-year overall survival was significantly higher in HBV-HCC recipients than in HCV-HCC recipients (76.65%, 56.61% and 49.10% vs. 64.59%, 42.78% and 39.20%, respectively; P<0.001). The corresponding tumor-free survival rates (63.55%, 47.37%, 40.99% vs. 56.84%, 38.04%, 35.66%, respectively) and hepatitis-free survival rates (75.49%, 54.84%, 47.34% vs. 63.87%, 42.15%, 39.33%, respectively) were both superior in HBV-HCC recipients (both P<0.001). Multivariate analyses identified hepatitis, preoperative alpha-fetoprotein (AFP) level, size of largest tumor, number of tumor nodules, TNM stage, vascular invasion and preoperative model for end-stage liver disease (MELD) score as independent predictors of overall, tumor-free and hepatitis-free survival.

**Conclusions:**

Survival outcomes after liver transplantation were significantly better in HBV-HCC patients than in HCV-HCC patients. This finding may be used to guide donor liver allocation in transplantation programs.

## Introduction

Hepatocellular carcinoma (HCC) resulting from chronic hepatitis B virus (HBV) and/or hepatitis C virus (HCV) infection is an important cause of liver disease worldwide [1], [2]. An estimated 400 million people in the world have HBV infection [3]. In China, HBV infection is the most important risk factor for the development of HCC. Approximately, 130 million people are HBV carriers [4], [5]. In addition, China has the highest incidence of HCC and accounts for 55% of all newly diagnosed HCC cases in the world [6]. HCV infects approximately 170 million individuals worldwide [7]. With an estimated prevalence of 1%–1.9% (approximately 13 million patients), China alone has more patients with HCV infection than all of Europe or the Americas [8]. Patients with hepatitis C are at risk of developing HCC at a rate of approximately 1%–3% per year [9].

Liver transplantation is a life-saving therapy for HBV-infected and HCV-infected patients with HCC. However, recurrence of HCC and hepatitis after liver transplantation has long been recognized in both hepatitis B and hepatitis C patients, which makes these infections the most important risk factors that determine prognosis and outcome after liver transplantation [10], [11], [12], [13].

The differences between HBV and HCV infections may result in different outcomes after liver transplantation. There are currently a number of studies that have been published regarding the different clinical characteristics of HBV-associated HCC (HBV-HCC) and HCV-associated HCC (HCV-HCC) [14], [15], [16], [17] and the transplantation outcomes for each condition [11], [12], [18], [19]. However, few studies have directly compared the survival outcomes of HBV-HCC and HCV-HCC or determined the impact of differences in HCC and/or hepatitis recurrence between HBV-HCC and HCV-HCC patients on overall survival. Given the current liver donor deficit, it seems important to understand the differences in the survival profiles of recipients with HBV-HCC and HCV-HCC, in order to better allocate liver grafts and to maximally benefit patients awaiting liver transplants.

We therefore analyzed the China Liver Transplant Registry (CLTR) data to evaluate clinical characteristics and compare survival rates after liver transplantation between recipients with HBV-HCC and those with HCV-HCC.

## Materials and Methods

### Ethics Statement

Ethical approval was obtained from Committee of Ethics in Biomedical Research of Zhejiang University. Written informed consent was obtained from all participants.

The research design was hospital-based and retrospective, with all cases being well evaluated. The research was approved by the CLTR (http://www.cltr.org/), which was initiated by the joint effort of the 20 most reputable liver transplant centers across the country in February 2005 and authorized as the only national liver transplant registry in Mainland China by the Ministry of Health in May 2008. Its database warehouse is administered by Center of Study for Liver Disease, Queen Mary Hospital, Department of Surgery, The University of Hong Kong.

### Objectives

The aim of this study was to compare the clinical characteristics and survival rates of HBV-HCC and HCV-HCC patients after liver transplantation in order to provide useful evidence for donor liver allocation.

### Participants

In all, 18,860 cases of liver transplantation were collected by the CLTR between January 1999 and December 2010 from 86 liver transplantation centers across China. The inclusion criteria for our study were as follows.

1. HCC: Pediatric liver transplantation patients and those who had undergone retransplantation, combined transplantation or liver transplantation for acute/fulminant liver failure were excluded. Patients with symptomatic HCC, pre-transplant HCC patients (or HCC patients waiting for liver transplantation) without symptoms, patients with HCC recurrence after primary liver resection, patients who underwent liver transplantation after interventional therapy for HCC, patients with other undefined tumors who underwent radiofrequency ablation (RFA) or transcatheter arterial chemoembolization (TACE) or other interventional therapies for HCC were included.
2. Type of liver transplantation: Both whole cadaveric donor liver transplant recipients and living donor liver transplant recipients were included.
3. Hepatitis B: Recipients diagnosed with “hepatitis B” according to etiology, and/or recipients who tested positive for “hepatitis B surface antigen (HBsAg)” and/or for “HBV-DNA on branched DNA (bDNA)” and/or “polymerase chain reaction (PCR)” assays were included.
4. Hepatitis C: recipients diagnosed with hepatitis C according to etiology were included.
5. HCC recurrence: Post-transplant patients with records for definite mentions of HCC recurrence or the date of first detection of HCC recurrence, and/or who underwent treatments for intra- and/or extrahepatic recurrence (intra-abdominal, chest recurrence and others) were included.
6. Hepatitis recurrence: The judgment of hepatitis B recurrence was on the basis of the following parameters included in the CLTR database during postoperative follow-up: a) histological change was marked with hepatitis B recurrence during postoperative follow-up; b) postoperative serological status changed from HBsAg or HBV-DNA (bDNA or PCR assay) negative to positive or there was the date of the transition. Post-transplant patients who were diagnosed with hepatitis C recurrence based on post-transplant liver biopsy were also included.

**Figure 1:**
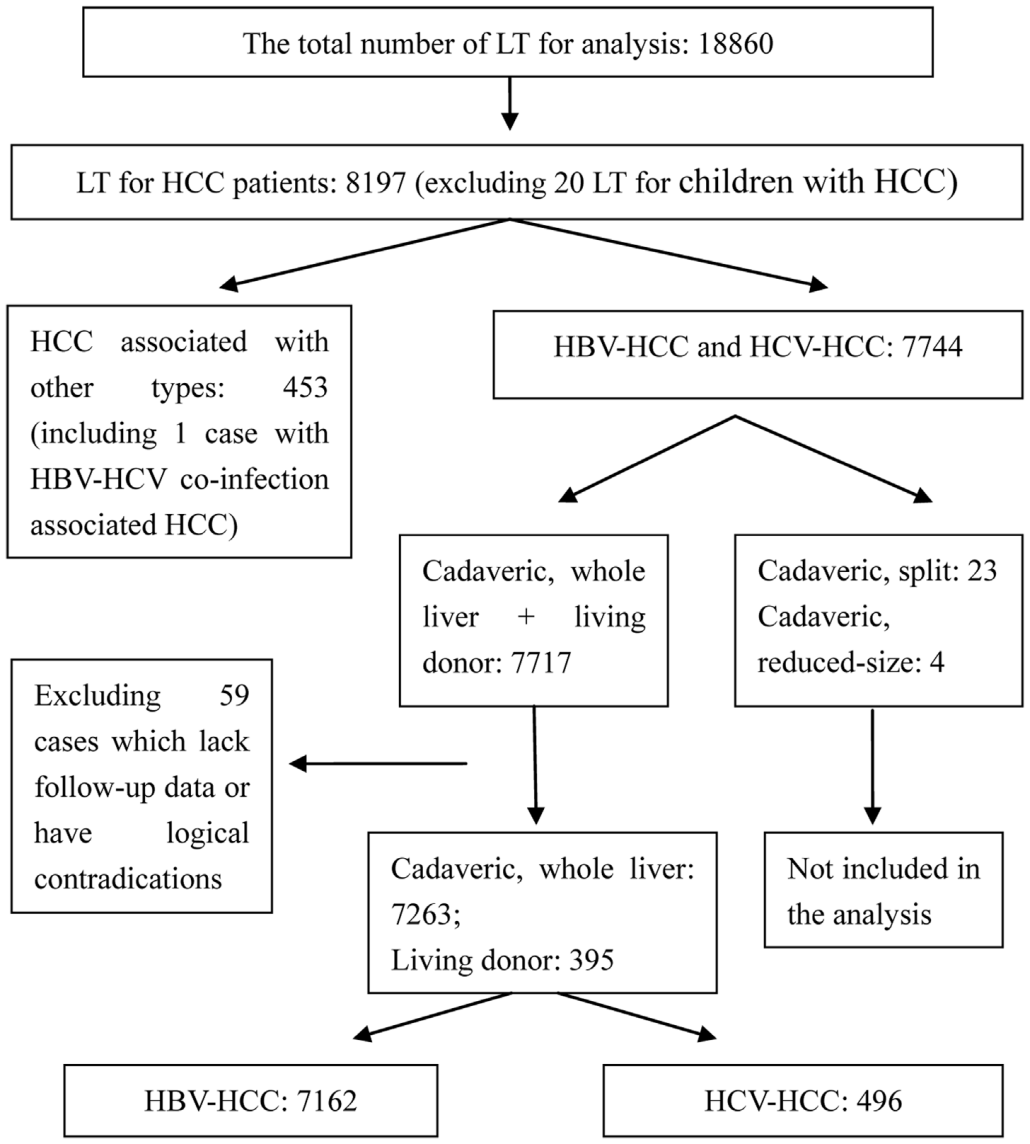
Flow chart of patient selection.

Using the above criteria, we excluded 11,202 patients from this study. We analyzed data pertaining to the remaining 7,658 patients who underwent liver transplantation for HBV-HCC or HCV-HCC (Fig. 1).

### Procedures

The 7,658 patients were divided into two groups according to the type of viral infection: the HBV-HCC group with 7,162 patients and the HCV-HCC group with 496 patients.

We compared the following clinical parameters between the two groups: age, gender, vascular invasion, downstaging, number of tumor nodules, size of largest tumor nodule, sum of tumor diameters and preoperative alpha fetoprotein (AFP) level. In addition, we compared the frequency of major postoperative complications between the two groups, including biliary and vascular complications, postoperative infections, renal failure and graft dysfunction.

The antiviral therapeutic schedules for hepatitis recurrence were mainly judged by the date of commencement of antiviral therapy with lamivudine, entecavir, adefovir, or telbivudine in the “postoperative data-recipient hepatitis serological status” column in the CLTR database. The above four antiviral drugs are all used to prevent hepatitis B. As for postoperative hepatitis C antiviral prevention programs, CLTR database does not include related drugs in a short time.

Patient survival was assessed using the Hangzhou criteria, which are as follows: patients without macrovascular invasion who have one of the two following items: (a) total tumor diameter less than or equal to 8 cm; (b) total tumor diameter more than 8 cm, with histopathologic grade I or II and preoperative AFP level less than or equal to 400 ng/ml, simultaneously. We have previously shown that the Hangzhou criteria are similar to the Milan criteria for defining good prognosis [20]. Of the 7,658 study patients, 3,009 (39.29%) met the Hangzhou criteria, with 38.75% patients (2,775/7,162) in the HBV-HCC group and 47.18% patients (234/496) in the HCV-HCC group. We calculated the 1-year, 3-year and 5-year overall, tumor-free and hepatitis-free survival rates from the operation date and compared these rates between the HBV-HCC and HCV-HCC groups, first for all patients and then for those who met the Hangzhou criteria.

The judgment for hospital mortality of the transplant patient is based on the postoperative information. In the CLTR database, the cases marked with hospital mortality have following features: a) the last follow-up date = the date of hospital discharge = the date of death; and/or b) the last follow-up date = transplant date; and/or c) the last follow-up date is later than the hospital discharge date and transplant date, but only within a few days. The 1-year, 3-year and 5-year overall, tumor-free and hepatitis-free survival rates were also analyzed after excluding cases of hospital mortality.

### Statistical Methods

Descriptive statistics were expressed as median (interquartile range). The chi square test or Fisher test was used for univariate comparisons where appropriate. For univariate survival analysis, plots were created and comparisons were made using the Kaplan-Meier method. The multivariate Cox proportional hazards regression analysis was used to identify predictors of recipient survival. Differences were considered statistically significant at P≤0.05. All statistical analyses were performed using SAS software, version 9.2.

## Results

### Patient Profiles

The patient profiles and overall characteristics are listed in Table 1. Of the 7,658 study subjects, 7,162 had HBV-HCC and 496 had HCV-HCC. Significant differences in sex distribution were observed between the HBV-HCC and HCV-HCC groups; 91.01% of HBV-HCC recipients (6,518/7,162) were male, while only 81.85% of HCV-HCC recipients (406/496) were male (P<0.001). The median duration of follow-up was 12.43 months (range, 3.42–29.61 months) for all patients. The following parameters significantly differed between the HBV-HCC and HCV-HCC groups (differences shown as HBV-HCC vs. HCV-HCC): age, number of tumors (median 1, range 1–3 vs. median 2, range 1–4), size of largest tumor (median 4 cm, range 2.5–7.0 cm vs. median 3.3 cm, range 2.5–5.0 cm) and preoperative AFP level (median 142.66 ng/ml, range 14.45–1,000 ng/ml vs. median 55.78 ng/ml, range 11.95–590.15 ng/ml). However, no significant difference was observed in the sum of tumor diameters (median 5 cm, range 3–10 cm vs. median 5 cm, range 3–9.5 cm, P = 0.631). Downstaging treatments, including TACE, RFA, percutaneous ethanol injection, systemic chemotherapy and combination therapy, were more common in the HCV-HCC group (P<0.001). The incidence of vascular invasion did not significantly differ between the HBV-HCC and HCV-HCC groups (P = 0.471).

**Table 1:** Clinical characteristics of patients with viral hepatitis-associated HCC who underwent liver transplantation.

Characteristic	HBV-associated HCC (N = 7,162)	HCV-associated HCC (N = 496)	P-value
Gender (male/female)	6,518/642*	406/90	<0.001
Age (years), no. (%)			<0.001
18–30	130 (1.82)	2 (0.40)	
30–40	959 (13.39)	8 (1.61)	
40–50	2,563 (35.79)	72 (14.52)	
50–65	3,180 (44.40)	326 (65.73)	
≥65	330 (4.61)	88 (17.74)	
Number of tumors#, median (interquartile range)	1 (1,3)	2 (1–4)	<0.001
Diameter of largest tumor<$>\raster(70%)="rg1"<$>, median (interquartile range), cm	4 (2.5–7)	3.3 (2.5,5.0)	<0.001
Sum of tumor diameters▴, median (interquartile range), cm	5 (3,10)	5 (3,9.5)	0.631
Preoperative AFP level★, median (interquartile range), ng/ml	142.66 (14.45,1,000)	55.78 (11.95,590.15)	<0.001
Downstaging, no. (%)	2,541 (35.48)	247 (49.80)	<0.001
TACE	1,680 (23.46)	146 (29.44)	
RFA	271 (3.78)	46 (9.27)	
Systemic chemotherapy	109 (1.52)	6 (1.21)	
Percutaneous ethanol injection	40 (0.56)	9 (1.81)	
Combination treatment	441 (6.16)	40 (8.06)	
Vascular invasion, no. (%)	2,087 (29.14)	137 (27.62)	0.471
Venous invasion	1,555 (21.71)	101 (20.36)	
Portal vein intrahepatic branch	934 (13.04)	67 (13.51)	
Portal vein right or left branch	853 (11.91)	35 (7.06)	
Main portal vein	844 (11.78)	47 (9.48)	
Hepatic vein	240 (3.35)	14 (2.82)	
Inferior vena cava	76 (1.06)	7 (1.41)	

* Gender was not specified in two cases;

# 1145 cases reported with missing nodules data and 144 cases with abnormal nodules were deleted;

^<$>\raster(70%)="rg1"<$>^790 cases reported with missing size data and 301 cases with abnormal size were deleted;

▴ 2352 cases reported with missing data and 286 cases with abnormal data were deleted;

★ 746 cases reported with missing AFP and 179 cases with abnormal AFP were deleted.

Abbreviations: HCC, hepatocellular carcinoma; AFP, alpha fetoprotein; TACE, transcatheter arterial chemoembolization; RFA, Radiofrequency ablation.

### Postoperative Complications

No significant differences existed in the rates of postoperative biliary and vascular complications and infections. However, compared to the HBV-HCC recipients, the HCV-HCC recipients showed a high incidence of renal failure and graft dysfunction (P = 0.015 and P = 0.006, respectively; Table 2).

**Table 2:** Postoperative complications in patients with viral hepatitis-associated HCC who underwent liver transplantation.

Postoperative Complications, no. (%)	HBV-associated HCC (N = 7,162)	HCV-associated HCC (N = 496)	P-value
Postoperative infections<$>\raster(70%)="rg1"<$>	1848 (25.80)	146 (29.44)	0.081
Biliary complications§	845 (11.80)	55 (11.09)	0.718
Renal failure★	212 (2.96)	25 (5.04)	0.015
Graft dysfunction*	147 (2.05)	20 (4.03)	0.006
Vascular complications♦	246 (3.43)	17 (3.43)	1

^<$>\raster(70%)="rg1"<$>^Postoperative infections include pulmonary infection, catheter-related sepsis, urinary tract infection, wound infection and opportunistic infections.

§ Biliary complications include anastomotic biliary strictures, intrahepatic biliary strictures and bile leakage.

★ Renal failure includes chronic renal failure, acute renal failure and uremia (excluding renal failure accompanied by hypertension and neonatal uremia).

* Graft dysfunction includes primary graft non-function and/or delayed graft function.

^♦^Vascular complications include hepatic artery embolism, portal vein embolism, portal vein stenosis/pylethrombosis, hepatic vein/inferior vena cava stenosis/embolism.

Abbreviation: HCC, hepatocellular carcinoma; HBV, hepatitis B virus; HCV, hepatitis C virus.

### Survival Analysis

In all recipients, the 1-year, 3-year and 5-year overall survival rates were 75.92%, 55.84% and 48.53%, respectively, and the corresponding tumor-free survival rates were 63.13%, 46.86% and 40.67%.

HCC recurrence was detected in 25.27% of all patients (1,935/7,658), 26.39% of HBV-HCC patients (1,890/7,162) and 9.07% of HCV-HCC patients (45/496) (P<0.001). In all, 186 recipients developed hepatitis recurrence, including 173 patients with hepatitis B and 13 with hepatitis C (P = 0.774). In the HBV-HCC group, HCC recurrence was significantly more common in patients with recurrent hepatitis B (75/173, 43.35%) than in patients without recurrent hepatitis B (1,815/6,989, 25.97%; P<0.001). However, in the HCV-HCC group, no significant difference was observed in the incidence of HCC recurrence between patients with recurrent hepatitis C (2/13, 15.38%) and those without recurrent hepatitis C (43/483, 8.90%; P = 0.422).

The 1-year, 3-year and 5-year overall survival rates (76.65%, 56.61% and 49.10%, respectively) were significantly higher in the HBV-HCC group than in the HCV-HCC group (64.59%, 42.78% and 39.20%, respectively; P<0.001). The corresponding tumor-free survival rates (63.55%, 47.37% and 40.99%) were also significantly higher in the HBV-HCC group than in the HCV-HCC group (56.84%, 38.04% and 35.66%; P<0.001). In addition, the corresponding hepatitis-free survival rates were significantly higher in the HBV-HCC group (75.49%, 54.84% and 47.34%) than in the HCV-HCC group (63.87%, 42.15%, and 39.33%; P<0.001).

Pre-transplant status, as judged by number of tumor nodules, size of largest tumor, preoperative AFP level and neoadjuvant treatment, significantly differed between the HBV-HCC and HCV-HCC groups, and may have influenced the survival rates. We therefore repeated the survival analysis for only recipients who satisfied the Hangzhou criteria. HCC recurrence was observed in 16.98% (511/3,009) of all patients who met the Hangzhou criteria, and 17.95% (498/2,775) of HBV-HCC patients and 5.56% (13/234) of HCV-HCC patients who met the Hangzhou criteria (P<0.0001). Moreover, hepatitis recurrence occurred in 80 (2.66%) recipients who met the Hangzhou criteria, including 69 (2.49%) in the HBV-HCC group and 11 (4.70%) in the HCV-HCC group (P = 0.043).

**Figure 2:**
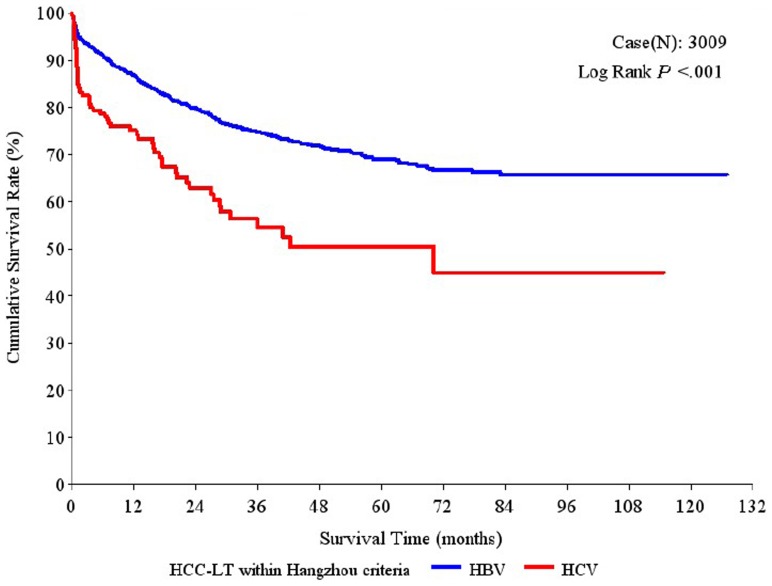
Overall survival rates after liver transplantation (LT) in patients with hepatitis B virus (HBV)-associated hepatocellular carcinoma (HCC) and hepatitis C virus (HCV)-associated HCC who met the Hangzhou criteria.

**Figure 3:**
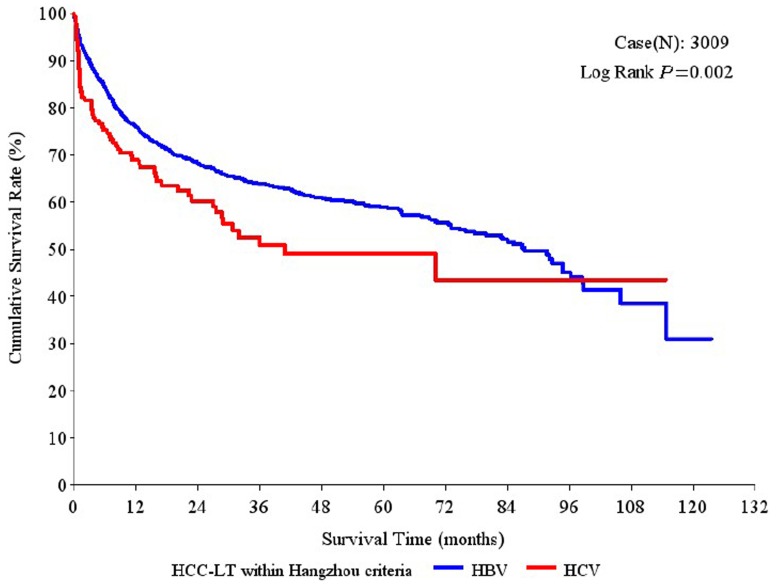
Tumor-free survival rates after liver transplantation (LT) in patients with hepatitis B virus (HBV)-associated hepatocellular carcinoma (HCC) and hepatitis C virus (HCV)-associated HCC who met the Hangzhou criteria.

**Figure 4:**
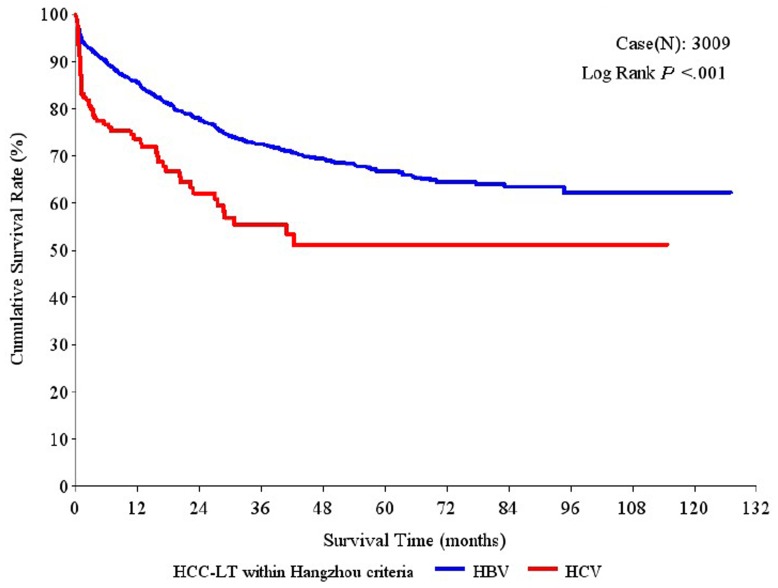
Hepatitis-free survival rates after liver transplantation (LT) in patients with hepatitis B virus (HBV)-associated hepatocellular carcinoma (HCC) and hepatitis C virus (HCV)-associated HCC who met the Hangzhou criteria.

Moreover, among the patients who met the Hangzhou criteria, the 1-year, 3-year and 5-year overall survival rates were significantly higher in the HBV-HCC group (86.89%, 74.71% and 68.99%, respectively) than in the HCV-HCC group (75.08%, 56.32% and 50.46%, respectively; P<0.001; Fig. 2). Similarly, compared to the HCV-HCC group, the HBV-HCC group had significantly higher 1-year, 3-year and 5-year tumor-free survival rates (75.98%, 63.93%, 58.85% vs. 68.89%, 52.55%, 48.92%, respectively; P = 0.002; Fig. 3) and hepatitis-free survival rates (85.52%, 72.40%, 66.69% vs. 73.62%, 55.39%, 51.12%, respectively; P<0.001; Fig. 4).

Hospital mortality was 4.56% in the entire cohort, 4.44% (318/7,162) in the HBV-HCC group and 6.25% (31/496) in the HCV-HCC group (P = 0.0616). Survival analysis was repeated after excluding cases of hospital mortality, and results similar to those shown above were obtained (data not shown).

### Multivariate Analysis

Although multiple predictors of survival were identified using univariate analysis (File S1), the important predictors for inferior overall, tumor-free and hepatitis-free survival included in the Cox regression model were as follows: hepatitis, preoperative AFP level ≥500 ng/ml, size of largest tumor >5 cm, number of tumor nodules >4, TNM stage IV, preoperative model for end-stage liver disease (MELD) score of 31–40 and vascular invasion (Table 3). In the HBV-HCC group, multivariate analysis revealed that all of the above parameters (except for hepatitis) were predictors of survival; in the HCV-HCC group, only number of tumor nodules >4, TNM stage IV and vascular invasion were associated with inferior overall, tumor-free and hepatitis-free survival, and none of these parameters reached statistical significance (File S2).

**Table 3:** Cox proportional hazards model.

				Overall survival				Tumor free survival				Hepatitis free survival			
				N = 4732<$>\raster(70%)="rg1"<$>				N = 4732<$>\raster(70%)="rg1"<$>				N = 4732<$>\raster(70%)="rg1"<$>			
Factor★	Group		Reference group	P	Hazard ratio	95% Confidence interval		P	Hazard ratio	95% Confidence interval		P	Hazard ratio	95% Confidence interval	
**Hepatitis**	**HBV**	**V.S.**	**HCV**	<.001	0.516	0.42	0.63	<.001	0.636	0.53	0.76	<.001	0.545	0.45	0.66
**Preoperative AFP**	**0–125**	**V.S.**	≥**500**	<.001	0.718	0.64	0.81	<.001	0.698	0.63	0.77	<.001	0.722	0.65	0.81
	**125–200**			0.296	0.882	0.70	1.12	0.002	0.719	0.58	0.89	0.168	0.848	0.67	1.07
	**200–500**			0.134	0.883	0.75	1.04	0.093	0.887	0.77	1.02	0.087	0.870	0.74	1.02
**Size of largest tumor**	≤**5 cm**	**V.S.**	**>5 cm**	<.001	0.696	0.62	0.78	<.001	0.665	0.60	0.73	<.001	0.711	0.64	0.80
**Number of tumor nodules**	≤**4**	**V.S.**	**>4**	<.001	0.756	0.67	0.86	<.001	0.766	0.68	0.86	<.001	0.781	0.69	0.89
**TNM staging**	**Stage I**	**V.S.**	**Stage IV**	<.001	0.469	0.37	0.57	<.001	0.469	0.37	0.57	<.001	0.489	0.39	0.61
	**Stage II**			<.001	0.532	0.43	0.63	<.001	0.532	0.43	0.63	<.001	0.537	0.45	0.65
	**Stage III**			0.017	0.820	0.70	0.97	0.046	0.865	0.75	1.00	0.029	0.834	0.71	0.98
**Vascular invasion**	**Yse**	**V.S.**	**No**	<.001	1.538	1.37	1.73	<.001	1.470	1.33	1.63	<.001	1.510	1.34	1.70
**Preoperative MELD score**	**6–20**	**V.S.**	**31–40**	<.001	0.512	0.40	0.66	<.001	0.591	0.47	0.75	<.001	0.543	0.42	0.70
	**21–30**			0.005	0.659	0.49	0.88	0.008	0.694	0.53	0.91	0.009	0.683	0.51	0.91

^<$>\scale 48%\raster="rg1"<$>^Cases in which data for any of the variables listed was missing (N = 2926) were excluded.

★ Adjusted for transplant year, recipient gender, recipient age and graft type (cadaveric/living).

## Discussion

HBV-HCC and HCV-HCC are the main indications for liver transplantation. The early outcomes of liver transplantation for viral hepatitis-related HCC were unsatisfactory [21]. With the introduction of the Milan criteria by Mazzaferro et al. [22], post-transplant survival rates in patients with HCC have become comparable to the rates in patients without HCC. However, the Milan criteria and other selection criteria regard patients with HBV-HCC and those with HCV-HCC as a homogeneous group, implying that donor livers can be allocated equally to both types of patients. However, HBV-associated hepatocarcinogenesis is quite different from that associated with HCV [2], [23], [24]. In addition, recurrent hepatitis after liver transplantation has been greatly reduced in patients with HBV infection [25], but not in patients with HCV infection [26], [27]. Therefore, transplantation outcomes may differ between patients with HBV-HCC and HCV-HCC. Although Mazzaferro et al. [22] included both HBV-HCC and HCV-HCC patients in their study, they did not separately analyze the survival of these two patient groups. Notably, among the 48 HCC patients in their study, 32 (66.7%) had HCV-HCC while only 11 (22.9%) had HBV-HCC. Therefore, the Milan criteria may favor HCV-HCC patients in terms of donor liver allocation. Bozorgzadeh et al. [28] suggested that the Milan criteria might be too restrictive when applied to patients without HCV infection. Yataco et al. [19], in their study, included HCC patients who did not meet the Milan criteria, and obtained excellent, long-term, post-transplant survival in patients with chronic HBV. So it is likely that we should take the different status of virus infection into consideration in decisions about donor liver allocation to HCC patients.

We found that the overall, tumor-free and hepatitis-free survival rates after liver transplantation were significantly worse in HCV-HCC patients than in HBV-HCC patients. In our patients, the pre-transplant status of recipients with HBV-HCC differed considerably from that of recipients with HCV-HCC. The HBV-HCC patients tended to be younger. Comparisons of HBV-HCC and HCV-HCC patients who underwent liver transplantations in the USA, Japan and Italy have revealed similar findings [14], [15], [16], [17]. This difference in age may reflect differences in the timing and sources of hepatitis infections. HBV infection usually occurs during infancy or early childhood because of perinatal or child-to-child spread, whereas HCV infections are usually acquired through blood transfusion or injectable drug use during adulthood. Also, virtually all HCV patients are already cirrhotic at the time of HCC development, whereas for HBV, up to 30% may not be cirrhotic. As cirrhosis takes time to develop, this will likely be another major contributor to the difference in age observed.

We also found a higher male/female ratio, greater size of largest tumor and higher AFP level in the HBV-HCC group, and a greater number of tumors per patient and downstaging treatments in the HCV-HCC group. These clinicobiological differences, which have also been observed in other studies [14], [16], [17], were largely attributable to differences in the mechanism of hepatocarcinogenesis between HBV and HCV. Studies have shown that HCV tends to cause chronic infections (10% of HBV cases vs. 60%–80% of HCV cases) [29], possibly because of immune evasion by HCV quasispecies resulting from high rates of replication errors [29], [30]. Moreover, HCV has a 10–20-fold higher propensity to promote cirrhosis than does HBV, with 5%–10% of hepatitis C patients developing liver cirrhosis after 10 years [29]. As an RNA virus, HCV cannot integrate into host genomes [29], and different gene expression profiles have been observed between hepatitis B and hepatitis C patients [31], [32]; these differences may lead to different protein characteristics between HBV-HCC and HCV-HCC patients [33]. The differences in viral proteins and the chemical nature of the genome will probably result in different molecular events during chronic hepatitis and hepatocarcinogenesis.

In our study, postoperative graft dysfunction was more common in the HCV-HCC group; this finding is consistent with the results of Waki et al. [34]. They analyzed liver graft survival in patients with different types of viral hepatitis, using data from the United Network Organ Sharing (UNOS), and observed that recipients with HBV monoinfection had the highest graft survival, at a 1-year survival of 85.3% and 10-year survival of 63.0%. In contrast, patients with HCV monoinfection had the lowest graft survival, at a 1-year survival of 82.9% and 10-year survival of 46.1%. They also concluded that patients with HCV monoinfection had a higher risk of graft loss. In our study, renal failure was more frequent in the HCV-HCC group. Burra et al. [35] reported that the median 1-year and 5-year glomerular filtration rate was significantly lower in HCV-positive patients than in HCV-negative patients. This finding may be largely attributable to HCV-induced glomerulonephritis. Although HBV is well-known to cause glomerulonephritis [36], Lee et al. [37] found no evidence of HBV-related immune complex glomerulonephritis on kidney biopsy in patients who underwent liver transplantation for HBV-related liver disease. The difference in the incidence of renal failure may also be related to the age differences between the HBV-HCC and HCV-HCC groups. Lee et al. [37] has shown that old age is a predictor for the development of chronic kidney disease after liver transplantation.

In our analysis, the overall, tumor-free and hepatitis-free survival rates were all significantly better in the HBV-HCC group, both when the entire cohort was considered and when only patients meeting the Hangzhou criteria were considered. These findings demonstrated that survival after liver transplantation did differ between recipients with HBV-HCC and those with HCV-HCC, regardless of differences in pre-transplant status. In addition, the multivariate analysis also identified type of viral hepatitis as an important predictor of survival, implying that the difference in virus infection has significantly affected post-transplant survival and recurrence.

The differences in survival between the HBV-HCC and HCV-HCC groups may largely attribute to two factors. First, differences in the prophylactic regimens for hepatitis recurrence between HBV and HCV might lead to differences in graft survival between these two groups of recipients, and therefore to different overall survival. Second, differences in the mechanisms of hepatocarcinogenesis between HBV and HCV may lead to different clinical profiles as mentioned above and affect post-transplantation recovery and HCC or hepatitis recurrence.

Our findings demonstrate that the differences in survival rates after liver transplantation between the HBV-HCC and HCV-HCC groups were attributable to not only differences in tumor recurrence rates but also differences in hepatitis recurrence rates. Hepatitis may recur because of intra- and extrahepatic viral reservoirs [38], whereas HCC recurrence might be caused by pretransplant or intra-operative spread of remnant tumor cells after a long time in a dormant state [39]. Both hepatitis recurrence and tumor recurrence can lead to graft failure and clinical decompensation, which impair recipient survival [12], [40], [41]. Nevertheless, the relationship between HCC recurrence and hepatitis recurrence after liver transplantation remains controversial. Some studies have shown that hepatitis recurrence is a risk factor for HCC recurrence, and that combination therapy for the prevention of hepatitis recurrence significantly improves overall and tumor-free survival following liver transplantation [42]. However, other studies have contradicted these findings, suggesting that HCC recurrence is a critical component for hepatitis recurrence [10], [43], [44] and that impaired immunity due to the cumulative corticosteroid dose and chemotherapy used for HCC recurrence lead to hepatitis recurrence [45]. Li et al. [46] observed a strong correlation between the timing of hepatitis recurrence and HCC recurrence. Our study demonstrated that in HBV-HCC patients, hepatitis B recurrence was associated with a higher incidence of HCC recurrence; however, a similar association was not found in the HCV-HCC patients. This may be because recurrent/metastatic HCC cells may support HBV replication and therefore be the source of HBV recurrence. Whereas in HCV, for those patients who are viraemic at the time of transplantation, almost all might have persistent infection; moreover, HCV-HCC patients who develop hepatitis C recurrence might die early, before they can develop HCC recurrence. Therefore the association with HCC is unlikely to be observed with HCV. However, the underlying mechanisms should be evaluated in future studies. So regarding these discrepancies between HBV-HCC and HCV-HCC patients, the developments of more accurate prediction algorithms based on meticulous evaluation of patient prognosis might be needed.

Our study has some limitations. First, as the study was not randomly assigned and included data from transplant centers all around China, the heterogenous nature of the data due to various criteria, definitions, as well as protocols among different centers - including definitions of HBV recurrence, antiviral therapy, post-transplant screening procedures for HCC, etc. - may have confounded our analysis. Second, because this was a clinical research study, which lacks the strong evidence of basic research studies, we could not fully explain the mechanisms underlying hepatitis or tumor recurrence that probably led to differences in survival between the HBV-HCC and HCV-HCC patients. Thirdly, the large number of missing data in the variable‘sum of tumor diameter’ may result in a potential bias in statistical analysis. This may be largely due to the factor that many recipients have diffuse tumor nodules, especially for those exceeding Hangzhou criteria, making it difficult to access the parameter accurately in reality. Fourthly, the relative short follow-up period (median of about1 year) makes it difficult to invest the long-term effects of different viruses on liver transplant recipients. Despite these limitations, our study represents the most comprehensive assessment of survival after liver transplantation in HBV-HCC and HCV-HCC patients to date, because we used data from the CLTR, which contains information on all liver transplants performed in China. Therefore, our results remain important in the development of donor liver allocation in patients with different types of viral hepatitis.

In conclusion, HCV-HCC patients have significantly worse overall, tumor-free and hepatitis-free survival after liver transplantation than HBV-HCC patients. Liver transplant recipients with HCV-HCC tended to be older and developed postoperative graft dysfunction and renal failure more frequently than recipients with HBV-HCC. Given the scarcity of donor livers, our findings might be considered to guide the development of future donor liver allocation protocols in transplantation programs, to take recipients with the same secondary diagnoses but with different primary diagnoses/etiologies into different considerations, in order to benefit those candidates who might obtain relatively superior survival rates more and thus improve overall survival in the total transplant population.

## Supporting Information

File S1 **Univariate survival analysis for patients with hepatocellular carcinoma.**
(DOC)

File S2 **Multivariate analysis for patients with hepatitis B virus or hepatitis C virus -associated hepatocellular carcinoma.**
(DOC)
