# Supplementary material for: Survival in Liver Transplant Recipients with Hepatitis B- or Hepatitis C-Associated Hepatocellular Carcinoma: The Chinese Experience from 1999 to 2010
Source: PLoS One. 2013 Apr 16;8(4):e61620. doi: 10.1371/journal.pone.0061620 (PMC3629024; doi:10.1371/journal.pone.0061620)
Supplement: File S1 — Univariate survival analysis for patients with hepatocellular carcinoma. (DOC) [file pone.0061620.s001.doc]

### **Table S1.** Overall survival rate after liver transplantation in patients with hepatocellular carcinoma (n = 7658)

| **Group** | | | **No.** | **Cumulative survival (%)** | | | **Log Rank**  ***P*** |
| --- | --- | --- | --- | --- | --- | --- | --- |
|  |  |  |  | **1-year** | **3-year** | **5-year** |  |
| **Tumor characteristics** | **New Edmondson grading** ^1^ | Well differentiated | 1477 | 80.99 | 64.88 | 59.37 | <.001 |
|  |  | Moderately differentiated | 2657 | 76.37 | 55.02 | 48.85 |  |
|  |  | Poorly differentiated | 479 | 67.03 | 45.54 | 37.03 |  |
|  |  | Undifferentiated | 17 | 65.88 | 65.88 | 32.94 |  |
|  | **TNM tumor staging for HCC ^2^** | Stage I | 1547 | 83.49 | 71.64 | 66.70 | <.001 |
|  |  | Stage II | 2131 | 81.81 | 64.05 | 58.05 |  |
|  |  | Stage III | 2004 | 67.14 | 39.72 | 28.81 |  |
|  |  | Stage IV | 477 | 59.56 | 28.08 | 16.52 |  |
|  | **Vascular invasion** | Yes | 2224 | 63.79 | 33.85 | 24.31 | <.001 |
|  |  | No | 5434 | 81.11 | 65.31 | 59.10 |  |
|  | **Size of largest tumor** ^3^**(cm)** | ≤5 | 4360 | 81.04 | 65.00 | 57.90 | <.001 |
|  |  | >5 | 2211 | 65.96 | 37.97 | 29.45 |  |
|  | **Number of tumour nodules** ^4^ | ≤4 | 4849 | 78.24 | 59.84 | 53.00 | <.001 |
|  |  | >4 | 936 | 66.51 | 40.66 | 30.69 |  |
| **Preoperative**  **status** | **Preoperative AFP ^5^(ng/mL)** | 0-125 | 3322 | 81.75 | 66.83 | 60.22 | <.001 |
|  |  | 125-200 | 328 | 78.12 | 60.01 | 53.06 |  |
|  |  | 200-500 | 774 | 79.05 | 56.38 | 48.05 |  |
|  |  | ≥500 | 2309 | 67.72 | 41.91 | 34.28 |  |
|  | **Preoperative MELD score**^6^ | 6-20 | 6171 | 77.90 | 57.30 | 50.42 | <.001 |
|  |  | 21-30 | 564 | 65.07 | 50.08 | 41.87 |  |
|  |  | 31-40 | 185 | 60.69 | 42.93 | 33.56 |  |
|  | **Preoperative Child score** ^7^ | 5-6 | 2229 | 81.03 | 58.09 | 51.02 | <.001 |
|  |  | 7-9 | 2332 | 76.66 | 56.71 | 47.81 |  |
|  |  | 10-15 | 1079 | 72.06 | 54.47 | 46.56 |  |

1. Cases for which new Edmondson grade was missing (n = 3028) were excluded.

2. Cases for which TNM stage was missing (n = 1499) were excluded.

3. Cases for the size of the largest tumor was missing (n = 1087) were excluded.

4. Cases with uncountable (n = 144) or unspecified (n = 1729) number of tumor nodules were excluded.

5. Cases for which preoperative alpha-fetoprotein (AFP) level was missing (n = 925) were excluded.

6. Cases for which preoperative model for end-stage liver disease (MELD) score was missing (n = 738) were excluded.

7. Cases for which preoperative Child score was missing (n = 2018) were excluded.

**Table S2.** Overall survival rate after liver transplantation in patients with hepatitis B virus-associated hepatocellular carcinoma (n = 7162)

|  | | | | | | | | |
| --- | --- | --- | --- | --- | --- | --- | --- | --- |
| **Group** | | | **No.** | **Cumulative survival (%)** | | | | **Log Rank**  ***P*** |
|  |  |  |  | **1-year** | | **3-year** | **5-year** |  |
| **Tumor characteristics** | **New Edmondson grading** ^1^ | Well differentiated | 1252 | | 83.40 | 67.31 | 61.90 | <.001 |
|  |  | Moderately differentiated | 2528 | | 77.03 | 55.75 | 49.45 |  |
|  |  | Poorly differentiated | 451 | | 67.58 | 45.39 | 36.40 |  |
|  |  | Undifferentiated | 17 | | 65.88 | 65.88 | 32.94 |  |
|  | **TNM tumor staging for HCC ^2^** | Stage I | 1420 | | 84.56 | 72.34 | 67.33 | <.001 |
|  |  | Stage II | 1972 | | 83.55 | 65.99 | 59.90 |  |
|  |  | Stage III | 1895 | | 67.15 | 40.36 | 29.17 |  |
|  |  | Stage IV | 477 | | 59.73 | 28.02 | 16.48 |  |
|  | **Vascular invasion** | Yes | 2087 | | 64.23 | 34.21 | 24.42 | <.001 |
|  |  | No | 5075 | | 81.99 | 66.30 | 59.95 |  |
|  | **Size of largest tumor** ^3^**(cm)** | ≤5 | 4003 | | 82.23 | 66.48 | 59.22 | <.001 |
|  |  | >5 | 2117 | | 66.22 | 38.24 | 29.55 |  |
|  | **Number of tumour nodules** ^4^ | ≤4 | 4520 | | 78.95 | 60.47 | 53.51 | <.001 |
|  |  | >4 | 848 | | 67.71 | 41.65 | 31.32 |  |
| **Preoperative**  **status** | **Preoperative AFP ^5^(ng/mL)** | 0-125 | 3048 | | 82.95 | 68.29 | 61.51 | <.001 |
|  |  | 125-200 | 307 | | 78.44 | 60.63 | 53.54 |  |
|  |  | 200-500 | 738 | | 79.84 | 57.45 | 48.83 |  |
|  |  | ≥500 | 2190 | | 67.91 | 41.94 | 34.18 |  |
|  | **Preoperative MELD score** ^6^ | 6-20 | 5744 | | 78.77 | 58.18 | 51.13 | <.001 |
|  |  | 21-30 | 527 | | 65.83 | 50.92 | 42.57 |  |
|  |  | 31-40 | 172 | | 61.52 | 42.54 | 31.35 |  |
|  | **Preoperative Child score** ^7^ | 5-6 | 2136 | | 80.93 | 58.17 | 51.07 | <.001 |
|  |  | 7-9 | 2203 | | 76.84 | 57.37 | 48.15 |  |
|  |  | 10-15 | 1007 | | 72. 60 | 54.60 | 46.59 |  |

1. Cases for which new Edmondson grade was missing (n = 2914) were excluded.

2. Cases for which TNM stage was missing (n = 1428) were excluded.

3. Cases for the size of the largest tumor was missing (n = 1042) were excluded.

4. Cases with uncountable (n = 135) or unspecified (n = 1659) number of tumor nodules were excluded.

5. Cases for which preoperative alpha-fetoprotein (AFP) level was missing (n = 879) were excluded.

6. Cases for which preoperative model for end-stage liver disease (MELD) score was missing (n = 719) were excluded.

7. Cases for which preoperative Child score was missing (n = 1816) were excluded.

**Table S3.** Overall survival rate after liver transplantation in patients with hepatitis C virus-associated hepatocellular carcinoma (n = 496)

|  | | | | | | | |
| --- | --- | --- | --- | --- | --- | --- | --- |
| **Group** | | | **No.** | **Cumulative survival (%)** | | | **Log Rank *P*** |
|  |  |  |  | **1-year** | **3-year** | **5-year** |  |
| **Tumor characteristics** | **New Edmondson grading** ^1^ | Well differentiated | 225 | 64.80 | 46.81 | 39.21 | .958 |
|  |  | Moderately differentiated | 129 | 62.57 | 38.85 | 36.07 |  |
|  |  | Poorly differentiated | 28 | 58.30 | 49.97 | 49.97 |  |
|  |  | Undifferentiated | - |  |  |  |  |
|  | **TNM tumor staging for HCC ^2^** | Stage I | 127 | 70.01 | 63.71 | 60.17 | .022 |
|  |  | Stage II | 159 | 57.77 | 35.37 | 30.40 |  |
|  |  | Stage III | 109 | 67.83 | 23.54 | 19.61 |  |
|  |  | Stage IV | 30 | 55.82 | - | - |  |
|  | **Vascular invasion** | Yes | 173 | 56.67 | 26.28 | 22.99 | .010 |
|  |  | No | 359 | 67.71 | 49.02 | 45.36 |  |
|  | **Size of largest tumor** ^3^**(cm)** | ≤5 | 357 | 66.45 | 45.00 | 40.47 | .817 |
|  |  | >5 | 94 | 58.26 | 23.18 | 23.18 |  |
|  | **Number of tumour nodules** ^4^ | ≤4 | 329 | 67.43 | 49.50 | 45.20 | .001 |
|  |  | >4 | 88 | 53.86 | 28.74 | 23.95 |  |
| **Preoperative**  **status** | **Preoperative AFP ^5^(ng/mL)** | 0-125 | 274 | 66.90 | 46.66 | 42.76 | .876 |
|  |  | 125-200 | 21 | 74.73 | 49.04 | - |  |
|  |  | 200-500 | 36 | 62.22 | 32.67 | 32.67 |  |
|  |  | ≥500 | 119 | 64.13 | 42.18 | 36.55 |  |
|  | **Preoperative MELD score** ^6^ | 6-20 | 427 | 65.33 | 43.63 | 39.64 | .186 |
|  |  | 21-30 | 37 | 48.87 | - | - |  |
|  |  | 31-40 | 13 | 48.95 | 48.95 | 48.95 |  |
|  | **Preoperative Child score** ^7^ | 5-6 | 93 | 83.84 | 55.31 | 48.40 | .329 |
|  |  | 7-9 | 129 | 73.57 | 42.78 | 42.78 |  |
|  |  | 10-15 | 72 | 64.41 | 54.67 | 49.21 |  |

1. Cases for which new Edmondson grade was missing (n =114) were excluded.

2. Cases for which TNM stage was missing (n = 71) were excluded.

3. Cases for the size of the largest tumor was missing (n = 45) were excluded.

4. Cases with uncountable (n = 9) or unspecified (n = 70) number of tumor nodules were excluded.

5. Cases for which preoperative alpha-fetoprotein (AFP) level was missing (n = 46) were excluded.

6. Cases for which preoperative model for end-stage liver disease (MELD) score was missing (n = 19) were excluded.

7. Cases for which preoperative Child score was missing (n = 202) were excluded.

**Table S4.** Tumor-free survival rate after liver transplantation in patients with hepatocellular carcinoma (n = 7658)

| **Group** | | | **No.** | **Tumor free survival (%)** | | | **Log Rank *P*** |
| --- | --- | --- | --- | --- | --- | --- | --- |
|  |  |  |  | **1-year** | **3-year** | **5-year** |  |
| **Tumor characteristics** | **New Edmondson grading** ^1^ | Well differentiated | 1477 | 70.73 | 57.05 | 51.47 | <.001 |
|  |  | Moderately differentiated | 2657 | 61.05 | 44.60 | 40.59 |  |
|  |  | Poorly differentiated | 479 | 47.94 | 32.98 | 27.20 |  |
|  |  | Undifferentiated | 17 | 58.56 | 48.80 | 24.40 |  |
|  | **TNM tumor staging for HCC ^2^** | Stage I | 1547 | 75.23 | 63.44 | 58.27 | <.001 |
|  |  | Stage II | 2131 | 70.41 | 54.84 | 49.98 |  |
|  |  | Stage III | 2004 | 49.25 | 30.57 | 23.17 |  |
|  |  | Stage IV | 477 | 39.60 | 18.23 | 9.45 |  |
|  | **Vascular invasion** | Yes | 2224 | 45.83 | 25.12 | 18.37 | <.001 |
|  |  | No | 5434 | 70.51 | 56.22 | 50.45 |  |
|  | **Size of largest tumor** ^3^**(cm)** | ≤5 | 4360 | 70.12 | 55.89 | 50.01 | <.001 |
|  |  | >5 | 2211 | 48.37 | 28.80 | 21.72 |  |
|  | **Number of tumour nodules** ^4^ | ≤4 | 4849 | 65.78 | 50.62 | 44.92 | <.001 |
|  |  | >4 | 936 | 49.12 | 31.27 | 25.40 |  |
| **Preoperative**  **status** | **Preoperative AFP ^5^(ng/mL)** | 0-125 | 3322 | 70.46 | 56.43 | 50.66 | <.001 |
|  |  | 125-200 | 328 | 67.76 | 52.96 | 47.69 |  |
|  |  | 200-500 | 774 | 64..87 | 47.47 | 41.29 |  |
|  | **Preoperative MELD score** ^6^ | ≥500 | 2309 | 51.16 | 32.98 | 26.99 | <.001 |
|  |  | 6-20 | 6171 | 63.97 | 47.50 | 41.99 |  |
|  |  | 21-30 | 564 | 56.55 | 43.17 | 34.78 |  |
|  | **Preoperative Child score** ^7^ | 31-40 | 185 | 50.46 | 38.52 | 30.16 | .044 |
|  |  | 5-6 | 2229 | 64.59 | 46.57 | 41.21 |  |
|  |  | 7-9 | 2332 | 61.35 | 46.15 | 39.52 |  |
|  |  | 10-15 | 1079 | 60.87 | 47.60 | 38.68 |  |

1. Cases for which new Edmondson grade was missing (n = 3028) were excluded.

2. Cases for which TNM stage was missing (n = 1499) were excluded.

3. Cases for which the size of the largest tumor was missing (n = 1087) were excluded.

4. Cases with uncountable (n = 144) or unspecified (n = 1729) number of tumor nodules were excluded.

5. Cases for which preoperative alpha-fetoprotein (AFP) level was missing (n = 925) were excluded.

6. Cases for which preoperative model for end-stage liver disease (MELD) score was missing (n = 738) were excluded.

7. Cases for which preoperative Child score was missing (n = 2018) were excluded.

**Table S5.** Tumor-free survival rate after liver transplantation in patients with hepatitis B virus-associated hepatocellular carcinoma (n = 7162)

|  | | | | | | | |
| --- | --- | --- | --- | --- | --- | --- | --- |
| **Group** | | | **No.** | **Tumor free survival (%)** | | | **Log Rank *P*** |
|  |  |  |  | **1-year** | **3-year** | **5-year** |  |
| **Tumor characteristics** | **New Edmondson grading** ^1^ | Well differentiated | 1252 | 72.55 | 59.00 | 53.27 | <.001 |
|  |  | Moderately differentiated | 2528 | 61.40 | 44.92 | 40.86 |  |
|  |  | Poorly differentiated | 451 | 47.60 | 32.80 | 26.69 |  |
|  |  | Undifferentiated | 17 | 58.56 | 48.80 | 24.40 |  |
|  | **TNM tumor staging for HCC ^2^** | Stage I | 1420 | 76.19 | 63.99 | 58.72 | <.001 |
|  |  | Stage II | 1972 | 71.50 | 56.34 | 51.31 |  |
|  |  | Stage III | 1895 | 49.19 | 30.85 | 23.32 |  |
|  |  | Stage IV | 477 | 39.09 | 18.24 | 9.46 |  |
|  | **Vascular invasion** | Yes | 2087 | 45.85 | 25.48 | 18.51 | <.001 |
|  |  | No | 5075 | 71.12 | 56.82 | 50.88 |  |
|  | **Size of largest tumor** ^3^**(cm)** | ≤5 | 4003 | 70.95 | 56.94 | 50.89 | <.001 |
|  |  | >5 | 2117 | 48.49 | 29.01 | 21.77 |  |
|  | **Number of tumour nodules** ^4^ | ≤4 | 4520 | 66.20 | 51.04 | 45.19 | <.001 |
|  |  | >4 | 848 | 49.28 | 31.57 | 25.62 |  |
| **Preoperative**  **status** | **Preoperative AFP ^5^(ng/mL)** | 0-125 | 3048 | 71.32 | 57.52 | 51.51 | <.001 |
|  |  | 125-200 | 307 | 67.41 | 53.06 | 47.71 |  |
|  |  | 200-500 | 738 | 65.36 | 47.95 | 41.59 |  |
|  |  | ≥500 | 2190 | 51.16 | 33.04 | 26.92 |  |
|  | **Preoperative MELD score** ^6^ | 6-20 | 5744 | 64.46 | 48.09 | 42.42 | <.001 |
|  |  | 21-30 | 527 | 57.08 | 43.82 | 35.31 |  |
|  |  | 31-40 | 172 | 50.60 | 37.97 | 28.01 |  |
|  | **Preoperative Child score** ^7^ | 5-6 | 2136 | 64.34 | 46.53 | 41.19 | .067 |
|  |  | 7-9 | 2203 | 61.53 | 46.56 | 39.70 |  |
|  |  | 10-15 | 1007 | 60.84 | 47.40 | 38.36 |  |

1. Cases for which new Edmondson grade was missing (n = 2914) were excluded.

2. Cases for which TNM stage was missing (n = 1428) were excluded.

3. Cases for the size of the largest tumor was missing (n = 1042) were excluded.

4. Cases with uncountable (n = 135) or unspecified (n = 1659) number of tumor nodules were excluded.

5. Cases for which preoperative alpha-fetoprotein (AFP) level was missing (n = 879) were excluded.

6. Cases for which preoperative model for end-stage liver disease (MELD) score was missing (n = 719) were excluded.

7. Cases for which preoperative Child score was missing (n = 1816) were excluded.

**Table S6.** Tumor-free survival rate after liver transplantation in patients with hepatitis C virus-associated hepatocellular carcinoma (n = 496)

|  | | | | | | | |
| --- | --- | --- | --- | --- | --- | --- | --- |
| **Group** | | | **No.** | **Tumor free survival (%)** | | | **Log Rank *P*** |
|  |  |  |  | **1-year** | **3-year** | **5-year** |  |
| **Tumor characteristics** | **New Edmondson grading** ^1^ | Well differentiated | 225 | 64.80 | 46.81 | 39.21 | .958 |
|  |  | Moderately differentiated | 129 | 62.57 | 38.85 | 36.07 |  |
|  |  | Poorly differentiated | 28 | 58.30 | 49.97 | 49.97 |  |
|  |  | Undifferentiated | - |  |  |  |  |
|  | **TNM tumor staging for HCC ^2^** | Stage I | 127 | 70.01 | 63.71 | 60.17 | .022 |
|  |  | Stage II | 159 | 57.77 | 35.37 | 30.40 |  |
|  |  | Stage III | 109 | 67.83 | 23.54 | 19.61 |  |
|  |  | Stage IV | 30 | 55.82 | - | - |  |
|  | **Vascular invasion** | Yes | 173 | 56.67 | 26.28 | 22.99 | .010 |
|  |  | No | 359 | 67.71 | 49.02 | 45.36 |  |
|  | **Size of largest tumor** ^3^**(cm)** | ≤5 | 357 | 66.45 | 45.00 | 40.47 | .817 |
|  |  | >5 | 94 | 58.26 | 23.18 | 23.18 |  |
|  | **Number of tumour nodules** ^4^ | ≤4 | 329 | 67.43 | 49.50 | 45.20 | .001 |
|  |  | >4 | 88 | 53.86 | 28.74 | 23.95 |  |
| **Preoperative**  **status** | **Preoperative AFP ^5^(ng/mL)** | 0-125 | 274 | 66.90 | 46.66 | 42.76 | .876 |
|  |  | 125-200 | 21 | 74.73 | 49.04 | - |  |
|  |  | 200-500 | 36 | 62.22 | 32.67 | 32.67 |  |
|  |  | ≥500 | 119 | 64.13 | 42.18 | 36.55 |  |
|  | **Preoperative MELD score** ^6^ | 6-20 | 427 | 65.33 | 43.63 | 39.64 | .186 |
|  |  | 21-30 | 37 | 48.87 | - | - |  |
|  |  | 31-40 | 13 | 48.95 | 48.95 | 48.95 |  |
|  | **Preoperative Child score** ^7^ | 5-6 | 93 | 83.84 | 55.31 | 48.40 | .329 |
|  |  | 7-9 | 129 | 73.57 | 42.78 | 42.78 |  |
|  |  | 10-15 | 72 | 64.41 | 54.67 | 49.21 |  |

1. Cases for which new Edmondson grade was missing (n =114) were excluded.

2. Cases for which TNM stage was missing (n = 71) were excluded.

3. Cases for the size of the largest tumor was missing (n = 45) were excluded.

4. Cases with uncountable (n = 9) or unspecified (n = 70) number of tumor nodules were excluded.

5. Cases for which preoperative alpha-fetoprotein (AFP) level was missing (n = 46) were excluded.

6. Cases for which preoperative model for end-stage liver disease (MELD) score was missing (n = 19) were excluded.

7. Cases for which preoperative Child score was missing (n = 202) were excluded.

**Table S7.** Hepatitis-free survival rate after liver transplantation in patients with hepatocellular carcinoma (n = 7658)

| **Group** | | | **No.** | **Recurrence free survival (%)** | | | **Log Rank *P*** |
| --- | --- | --- | --- | --- | --- | --- | --- |
|  |  |  |  | **1-year** | **3-year** | **5-year** |  |
| **Tumor characteristics** | **New Edmondson grading** ^4^ | Well differentiated | 1477 | 80.21 | 63.45 | 58.68 | <.001 |
|  |  | Moderately differentiated | 2657 | 75.03 | 53.41 | 46.91 |  |
|  |  | Poorly differentiated | 479 | 65.72 | 43.09 | 33.25 |  |
|  |  | Undifferentiated | 17 | 59.59 | 59.59 | 29.79 |  |
|  | **TNM tumor staging for HCC ^5^** | Stage I | 1547 | 81.77 | 69.02 | 64.22 | <.001 |
|  |  | Stage II | 2131 | 80.81 | 61.80 | 55.36 |  |
|  |  | Stage III | 2004 | 65.83 | 38.56 | 28.28 |  |
|  |  | Stage IV | 477 | 59.66 | 27.75 | 13.58 |  |
|  | **Vascular invasion** | Yes | 2224 | 62.81 | 32.79 | 23.21 | <.001 |
|  |  | No | 5434 | 79.92 | 63.28 | 57.09 |  |
|  | **Size of largest tumor** ^6^**(cm)** | ≤5 | 4360 | 79.93 | 63.05 | 56.06 | <.001 |
|  |  | >5 | 2211 | 64.70 | 37.01 | 28.39 |  |
|  | **Number of tumour nodules** ^7^ | ≤4 | 4849 | 77.09 | 57.92 | 50.90 | <.001 |
|  |  | >4 | 936 | 65.57 | 39.86 | 30.71 |  |
| **Preoperative**  **status** | **Preoperative AFP ^11^(ng/mL)** | 0-125 | 3322 | 80.68 | 65.06 | 58.47 | <.001 |
|  |  | 125-200 | 328 | 77.78 | 58.81 | 53.11 |  |
|  |  | 200-500 | 774 | 77.96 | 54.39 | 46.77 |  |
|  |  | ≥500 | 2309 | 66.56 | 40.46 | 32.44 |  |
|  | **Preoperative MELD score** ^12^ | 6-20 | 6171 | 76.71 | 55.47 | 48.52 | <.001 |
|  |  | 21-30 | 564 | 64.06 | 48.95 | 41.36 |  |
|  |  | 31-40 | 185 | 60.71 | 41.88 | 32.68 |  |
|  | **Preoperative Child score** ^13^ | 5-6 | 2229 | 79.82 | 56.77 | 49.44 | <.001 |
|  |  | 7-9 | 2332 | 75.32 | 54.27 | 45.29 |  |
|  |  | 10-15 | 1079 | 70.82 | 52.59 | 44.89 |  |

1. Cases for which new Edmondson grade was missing (n = 3028) were excluded.

2. Cases for which TNM stage was missing (n = 1499) were excluded.

3. Cases for which the size of the largest tumor was missing (n = 1087) were excluded.

4. Cases with uncountable (n = 144) or unspecified (n = 1729) number of tumor nodules were excluded.

5. Cases for which preoperative alpha-fetoprotein (AFP) level was missing (n = 925) were excluded.

6. Cases for which preoperative model for end-stage liver disease (MELD) score was missing (n = 738) were excluded.

7. Cases for which preoperative Child score was missing (n = 2018) were excluded.

**Table S8.** Hepatitis-free survival rate after liver transplantation in patients with hepatitis B virus-associated hepatocellular carcinoma (n = 7162)

|  | | | | | | | |
| --- | --- | --- | --- | --- | --- | --- | --- |
| **Group** | | | **No.** | **Recurrence free survival (%)** | | | **Log Rank *P*** |
|  |  |  |  | **1-year** | **3-year** | **5-year** |  |
| **Tumor characteristics** | **New Edmondson grading** ^4^ | Well differentiated | 1252 | 82.57 | 65.87 | 61.09 | <.001 |
|  |  | Moderately differentiated | 2528 | 75.67 | 54.05 | 47.40 |  |
|  |  | Poorly differentiated | 451 | 66.28 | 42.95 | 32.51 |  |
|  |  | Undifferentiated | 17 | 59.59 | 59.59 | 29.79 |  |
|  | **TNM tumor staging for HCC ^5^** | Stage I | 1420 | 82.77 | 69.63 | 64.78 | <.001 |
|  |  | Stage II | 1972 | 82.52 | 63.72 | 57.04 |  |
|  |  | Stage III | 1895 | 65.83 | 39.12 | 28.60 |  |
|  |  | Stage IV | 477 | 59.91 | 27.69 | 13.55 |  |
|  | **Vascular invasion** | Yes | 2087 | 63.19 | 33.10 | 23.26 | <.001 |
|  |  | No | 5075 | 80.79 | 64.22 | 57.84 |  |
|  | **Size of largest tumor** ^6^**(cm)** | ≤5 | 4003 | 81.12 | 64.48 | 57.27 | <.001 |
|  |  | >5 | 2117 | 64.91 | 37.25 | 28.46 |  |
|  | **Number of tumour nodules** ^7^ | ≤4 | 4520 | 77.80 | 58.50 | 51.30 | <.001 |
|  |  | >4 | 848 | 66.67 | 40.88 | 31.40 |  |
| **Preoperative**  **Status** | **Preoperative AFP ^11^(ng/mL)** | 0-125 | 3048 | 81.83 | 66.38 | 59.62 | <.001 |
|  |  | 125-200 | 307 | 78.12 | 59.43 | 53.59 |  |
|  |  | 200-500 | 738 | 78.71 | 55.37 | 47.49 |  |
|  |  | ≥500 | 2190 | 66.81 | 40.56 | 32.30 |  |
|  | **Preoperative MELD score** ^12^ | 6-20 | 5744 | 77.54 | 56.25 | 49.10 | <.001 |
|  |  | 21-30 | 527 | 64.76 | 49.74 | 42.03 |  |
|  |  | 31-40 | 172 | 61.52 | 41.41 | 30.44 |  |
|  | **Preoperative Child score** ^13^ | 5-6 | 2136 | 79.79 | 56.94 | 49.44 | <.001 |
|  |  | 7-9 | 2203 | 75.40 | 54.80 | 45.51 |  |
|  |  | 10-15 | 1007 | 71.29 | 52.63 | 44.85 |  |

1. Cases for which new Edmondson grade was missing (n = 2914) were excluded.

2. Cases for which TNM stage was missing (n = 1428) were excluded.

3. Cases for the size of the largest tumor was missing (n = 1042) were excluded.

4. Cases with uncountable (n = 135) or unspecified (n = 1659) number of tumor nodules were excluded.

5. Cases for which preoperative alpha-fetoprotein (AFP) level was missing (n = 879) were excluded.

6. Cases for which preoperative model for end-stage liver disease (MELD) score was missing (n = 719) were excluded.

7. Cases for which preoperative Child score was missing (n = 1816) were excluded.

**Table S9.** Hepatitis-free survival rate after liver transplantation in patients with hepatitis C virus-associated hepatocellular carcinoma (n = 496)

|  | | | | | | | |
| --- | --- | --- | --- | --- | --- | --- | --- |
| **Group** | | | **No.** | **Recurrence free survival (%)** | | | **Log Rank *P*** |
|  |  |  |  | **1-year** | **3-year** | **5-year** |  |
| **Tumor characteristics** | **New Edmondson grading** ^3^ | Well differentiated | 225 | 64.62 | 45.47 | 40.00 | .971 |
|  |  | Moderately differentiated | 129 | 61.80 | 39.34 | 36.53 |  |
|  |  | Poorly differentiated | 28 | 56.14 | 46.79 | 46.79 |  |
|  |  | Undifferentiated | - |  |  |  |  |
|  | **TNM tumor staging for HCC ^4^** | Stage I | 127 | 69.27 | 62.84 | 59.14 | .028 |
|  |  | Stage II | 159 | 57.16 | 33.38 | 30.60 |  |
|  |  | Stage III | 109 | 66.78 | 23.90 | 19.92 |  |
|  |  | Stage IV | 30 | 54.60 | - | - |  |
|  | **Vascular invasion** | Yes | 173 | 56.67 | 26.28 | 22.99 | .017 |
|  |  | No | 359 | 66.75 | 47.90 | 45.30 |  |
|  | **Size of largest tumor** ^5^**(cm)** | ≤5 | 357 | 65.46 | 43.89 | 40.31 | .930 |
|  |  | >5 | 94 | 58.59 | 23.31 | 23.31 |  |
|  | **Number of tumour nodules** ^6^ | ≤4 | 329 | 66.26 | 48.62 | 45.53 | .001 |
|  |  | >4 | 88 | 54.21 | 27.67 | 23.06 |  |
| **Preoperative**  **status** | **Preoperative AFP ^10^(ng/mL)** | 0-125 | 274 | 66.52 | 46.94 | 43.01 | .844 |
|  |  | 125-200 | 21 | 74.21 | 48.70 | - |  |
|  |  | 200-500 | 36 | 61.86 | 32.47 | 32.47 |  |
|  |  | ≥500 | 119 | 61.74 | 38.95 | 35.96 |  |
|  | **Preoperative MELD score** ^11^ | 6-20 | 427 | 64.84 | 43.38 | 40.27 | .212 |
|  |  | 21-30 | 37 | 48.87 | - | - |  |
|  |  | 31-40 | 13 | 48.95 | 48.95 | 48.95 |  |
|  | **Preoperative Child score** ^12^ | 5-6 | 93 | 80.58 | 51.29 | 47.63 | .431 |
|  |  | 7-9 | 129 | 72.52 | 42.95 | 42.95 |  |
|  |  | 10-15 | 72 | 64.18 | 54.40 | 48.96 |  |

1. Cases for which new Edmondson grade was missing (n =114) were excluded.

2. Cases for which TNM stage was missing (n = 71) were excluded.

3. Cases for the size of the largest tumor was missing (n = 45) were excluded.

4. Cases with uncountable (n = 9) or unspecified (n = 70) number of tumor nodules were excluded.

5. Cases for which preoperative alpha-fetoprotein (AFP) level was missing (n = 46) were excluded.

6. Cases for which preoperative model for end-stage liver disease (MELD) score was missing (n = 19) were excluded.

7. Cases for which preoperative Child score was missing (n = 202) were excluded.
