# Supplementary material for: Survival in Liver Transplant Recipients with Hepatitis B- or Hepatitis C-Associated Hepatocellular Carcinoma: The Chinese Experience from 1999 to 2010
Source: PLoS One. 2013 Apr 16;8(4):e61620. doi: 10.1371/journal.pone.0061620 (PMC3629024; doi:10.1371/journal.pone.0061620)
Supplement: File S2 — Multivariate analysis for patients with hepatitis B virus or hepatitis C virus -associated hepatocellular carcinoma. (DOC) [file pone.0061620.s002.doc]

**Table S1.** Overall survival rate after liver transplantation in patients with hepatitis B virus-associated hepatocellular carcinoma (n = 4390^1^)

| **Factor ^2^** | **Group** |  | **Reference group** | ***P*** | **Hazard ratio** | **95% Confidence interval** | |
| --- | --- | --- | --- | --- | --- | --- | --- |
| **Preoperative AFP** | **0-125** | **V.S.** | ≥**500** | <.001 | 0.667 | 0.59 | 0.75 |
|  | **125-200** |  |  | 0.154 | 0.836 | 0.65 | 1.07 |
|  | **200-500** |  |  | 0.070 | 0.857 | 0.72 | 1.01 |
| **Size of largest tumor** | ≤**5 cm** | **V.S.** | **>5 cm** | <.001 | 0.657 | 0.58 | 0.74 |
| **Number of tumor nodules** | ≤**4** | **V.S.** | **>4** | <.001 | 0.733 | 0.64 | 0.84 |
| **TNM**  **staging** | **Stage I** | **V.S.** | **Stage IV** | <.001 | 0.464 | 0.37 | 0.58 |
|  | **Stage II** |  |  | <.001 | 0.515 | 0.42 | 0.63 |
|  | **Stage III** |  |  | 0.033 | 0.833 | 0.70 | 0.99 |
| **Vascular invasion** | **Yse** | **V.S.** | **No** | <.001 | 1.546 | 1.37 | 1.75 |
| **Preoperative MELD score** | **6-20** | **V.S.** | **31-40** | <.001 | 0.432 | 0.33 | 0.56 |
|  | **21-30** |  |  | 0.005 | 0.549 | 0.41 | 0.74 |

1. Cases in which data for any of the variables listed was missing (n = 2772) were excluded.
2. Adjusted for transplant year, recipient gender, recipient age and graft type (cadaveric/living).

**Table S2.** Tumor-free survival rate after liver transplantation in patients with hepatitis B virus-associated hepatocellular carcinoma (n = 4390^1^)

| **Factor ^2^** | **Group** |  | **Reference group** | ***P*** | **Hazard ratio** | **95% Confidence interval** | |
| --- | --- | --- | --- | --- | --- | --- | --- |
| **Preoperative AFP** | **0-125** | **V.S.** | ≥**500** | <.001 | 0.668 | 0.60 | 0.74 |
|  | **125-200** |  |  | 0.002 | 0.710 | 0.57 | 0.88 |
|  | **200-500** |  |  | 0.072 | 0.877 | 0.76 | 1.01 |
| **Size of largest tumor** | ≤**5 cm** | **V.S.** | **>5 cm** | <.001 | 0.641 | 0.58 | 0.71 |
| **Number of tumor nodules** | ≤**4** | **V.S.** | **>4** | <.001 | 0.745 | 0.66 | 0.84 |
| **TNM**  **staging** | **Stage I** | **V.S.** | **Stage IV** | <.001 | 0.536 | 0.44 | 0.65 |
|  | **Stage II** |  |  | <.001 | 0.585 | 0.49 | 0.69 |
|  | **Stage III** |  |  | 0.057 | 0.869 | 0.75 | 1.00 |
| **Vascular invasion** | **Yse** | **V.S.** | **No** | <.001 | 1.458 | 1.31 | 1.62 |
| **Preoperative MELD score** | **6-20** | **V.S.** | **31-40** | <.001 | 0.525 | 0.41 | 0.67 |
|  | **21-30** |  |  | <.001 | 0.611 | 0.46 | 0.81 |

1. Cases in which data for any of the variables listed was missing (n = 2772) were excluded.
2. Adjusted for transplant year, recipient gender, recipient age and graft type (cadaveric/living).

**Table S3.** Hepatitis-free survival rate after liver transplantation in patients with hepatitis B virus-associated hepatocellular carcinoma (n = 4390^1^)

| **Factor ^2^** | **Group** |  | **Reference group** | ***P*** | **Hazard ratio** | **95% Confidence interval** | |
| --- | --- | --- | --- | --- | --- | --- | --- |
| **Preoperative AFP** | **0-125** | **V.S.** | ≥**500** | <.001 | 0.677 | 0.60 | 0.76 |
|  | **125-200** |  |  | 0.080 | 0.804 | 0.63 | 1.03 |
|  | **200-500** |  |  | 0.045 | 0.846 | 0.72 | 1.00 |
| **Size of largest tumor** | ≤**5 cm** | **V.S.** | **>5 cm** | <.001 | 0.675 | 0.60 | 0.76 |
| **Number of tumor nodules** | ≤**4** | **V.S.** | **>4** | <.001 | 0.762 | 0.67 | 0.87 |
| **TNM**  **staging** | **Stage I** | **V.S.** | **Stage IV** | <.001 | 0.495 | 0.40 | 0.62 |
|  | **Stage II** |  |  | <.001 | 0.529 | 0.43 | 0.64 |
|  | **Stage III** |  |  | 0.052 | 0.848 | 0.72 | 1.00 |
| **Vascular invasion** | **Yse** | **V.S.** | **No** | <.001 | 1.523 | 1.35 | 1.72 |
| **Preoperative MELD score** | **6-20** | **V.S.** | **31-40** | <.001 | 0.466 | 0.36 | 0.60 |
|  | **21-30** |  |  | <.001 | 0.579 | 0.43 | 0.78 |

1. Cases in which data for any of the variables listed was missing (n = 2772) were excluded.
2. Adjusted for transplant year, recipient gender, recipient age and graft type (cadaveric/living).

**Table S4.** Overall survival rate after liver transplantation in patients with hepatitis C virus-associated hepatocellular carcinoma (n = 342^1^)

| **Factor ^2^** | **Group** |  | **Reference group** | ***P*** | **Hazard ratio** | **95% Confidence interval** | |
| --- | --- | --- | --- | --- | --- | --- | --- |
| **Number of tumor nodules** | ≤**4** | **V.S.** | **>4** | 0.052 | 0.616 | 0.38 | 1.00 |
| **TNM**  **staging** | **Stage I** | **V.S.** | **Stage IV** | 0.096 | 0.480 | 0.20 | 1.34 |
|  | **Stage II** |  |  | 0.183 | 0.589 | 0.27 | 1.28 |
|  | **Stage III** |  |  | 0.209 | 0.612 | 0.28 | 1.32 |
| **Vascular invasion** | **Yse** | **V.S.** | **No** | 0.967 | 1.010 | 0.63 | 1.62 |

1. Cases in which data for any of the variables listed was missing (n = 154) were excluded.
2. Adjusted for transplant year, recipient gender, recipient age and graft type (cadaveric/living).

**Table S5.** Tumor-free survival rate after liver transplantation in patients with hepatitis C virus-associated hepatocellular carcinoma (n = 342^1^)

| **Factor ^2^** | **Group** |  | **Reference group** | ***P*** | **Hazard ratio** | | **95% Confidence interval** | | |  |
| --- | --- | --- | --- | --- | --- | --- | --- | --- | --- | --- |
| **Number of tumor nodules** | ≤**4** | **V.S** | **>4** | 0.250 | | 0.753 | | 0.46 | 1.22 | |
| **TNM**  **staging** | **Stage I** | **V.S** | **Stage IV** | 0.150 | | 0.559 | | 0.25 | 1.24 | |
|  | **Stage II** |  |  | 0.259 | | 0.657 | | 0.32 | 1.36 | |
|  | **Stage III** |  |  | 0.201 | | 0.625 | | 0.30 | 1.29 | |
| **Vascular invasion** | **Yse** | **V.S** | **No** | 0.225 | | 1.323 | | 0.84 | 2.08 | |

1. Cases in which data for any of the variables listed was missing (n = 154) were excluded.
2. Adjusted for transplant year, recipient gender, recipient age and graft type (cadaveric/living).

**Table S6.** Hepatitis-free survival rate after liver transplantation in patients with hepatitis C virus-associated hepatocellular carcinoma (n = 342^1^)

| **Factor ^2^** | **Group** |  | **Reference group** | ***P*** | **Hazard ratio** | **95% Confidence interval** | |
| --- | --- | --- | --- | --- | --- | --- | --- |
| **Number of tumor nodules** | ≤**4** | **V.S** | **>4** | 0.070 | 0.636 | 0.39 | 1.04 |
| **TNM**  **staging** | **Stage I** | **V.S** | **Stage IV** | 0.096 | 0.481 | 0.20 | 1.14 |
|  | **Stage II** |  |  | 0.171 | 0.581 | 0.27 | 1.26 |
|  | **Stage III** |  |  | 0.221 | 0.620 | 0.29 | 1.33 |
| **Vascular invasion** | **Yse** | **V.S** | **No** | 0.917 | 0.975 | 0.61 | 1.56 |

1. Cases in which data for any of the variables listed was missing (n = 154) were excluded.
2. Adjusted for transplant year, recipient gender, recipient age and graft type (cadaveric/living).
